# The hidden vulnerability of homelessness in the COVID-19 pandemic: Perspectives from India

**DOI:** 10.1177/0020764020922890

**Published:** 2020-05-14

**Authors:** Debanjan Banerjee, Prama Bhattacharya

**Affiliations:** 1 Department of Psychiatry, National Institute of Mental Health and Neurosciences (NIMHANS), Bangalore, India; 2 Department of Humanities and Social Sciences, Indian Institute of Technology Kanpur, Kanpur, India

The past three months have marked the beginning of a global public health crisis. Coronavirus disease 2019 (COVID-19) caused by the novel coronavirus SARS-CoV-2 has emerged into a pandemic within 70 days of its initiation at the Wuhan province of China. Nearly 1.4 million has been affected globally, and 67,000 have succumbed to the infection so far, numbers rising as we write (World Health Organization (WHO) COVID-19 Situation Report, as on 8 April 2020). Borders are being shut down internationally, economies slashed, and massive travel restrictions imposed worldwide to contain the outbreak. Billions of people are isolated at their residences, waiting for the situation to stabilize. While the global public health agencies like the World Health Organization (WHO) and the Centers for Disease Control and Prevention (CDC) struggle for measures against the outbreak, precautionary strategies are the current mainstay.

Vulnerability is higher in the elderly, immunocompromised, malnourished and in people with pre-existing pulmonary and other systemic illnesses (Guo et al., 2020). Certain marginalized sections of the society, especially in countries with inadequate public health measures, can have a combination of all these factors along with psycho-social stressors, which make them uniquely at risk to any pandemic. These populations are at a public health crisis as a whole, are especially sensitive in global crises such as this.

The implications of an infection like this with significant public health burden can be immense for developing countries like India with age-old pandemic preparation policies. The first COVID-19 case in India got reported only about a month ago, which quickly escalated to a three-digit count in 2 days, with more than a thousand affected at present threatening a community transmission (Ministry of Health and Family Welfare, Government of India, n.d.). Cases when quickly escalated can cause significant problems for a already populous country like India, with a significant burden on the health-care resources. Competition for health-care, mass hysteria, misinformation and public fear add to this burden, posing more pertinent threats than the virus itself. While the government has decided an all-nation lockdown for 21 days to contain the spread, let us glance at the challenges faced by a selectively vulnerable population in India, the homeless, and how they contribute to the overall public health risk.

## Pandemics and homelessness: the problem statement

Pandemics are not just medical phenomena. They have immense psycho-social implications, affecting society at large. Homeless individuals, considered to be an ‘invisible burden’ to the society, are often not included in the country census, lacking accountability and responsibility for the respective administrative authorities. It has been documented during the earlier pandemics of SARS and Influenza that the homeless population poses unique vulnerabilities to themselves and public health. Rate of spread of infection, numbers affected, and mortality were all higher in them with a minimal percentage of detection and treatment (Leung et al., 2008). It had led to the development of various pandemic planning guides for the homeless and housing service providers, the implementation of which continue to be sketchy (Centers for Disease Control and Prevention, 2009). Accountability and early preparedness for this vulnerable population have been mentioned as key measures in such guides. Administrative sensitization towards the same however, is unfortunately lacking in many of the developing countries. It has been mentioned that COVID-19 also poses similar threats to the homeless with urgent housing, sanitation, and quarantine measures needed to contain the spread among them (Tsai & Wilson, 2020). Even though numbers and graphs get displayed every day about the rising viral spread, the homeless population is often not clearly represented doubtfully as their numbers are often incorrectly estimated. In the search for information during pandemics, their number is often forgotten and thus neglected from the consequent policies.

## Homelessness in India

Although widely considered to be a ‘social evil’, homelessness is more prevalent and neglected in low- and middle-income countries like India. Already a socio-culturally diverse and populated country, it houses a significant proportion of the world’s homeless population. As per the Census 2011, there are 1.77 million homeless individuals in the country, accounting for 0.15 percent of the total population (Goel & Chowdhary, 2017). However, the estimated numbers are mostly inconsistent. For example, in the capital city of Delhi, the same 2011 Census reported 46,724 homeless persons, which was 88,410 as per the Indo-Global Social Service Society and increased to 1,50,000 when counted by the Delhi Development Authority (DDA) during the same period (Zufferey & Yu, 2017). Few critical reasons for this are the very definition of homelessness, temporary living spaces, frequently migrating population, and lackluster attitude of the authorities. There are also a high number of street children and mentally ill, invariably as a part of the ‘homeless’ crowd. A family of four members has an average of five homeless generations in India (Goel & Chowdhary, 2017), which itself highlights the importance and vulnerability of this group in India’s public health. Due to the lack of numbers and accountability, adequate public health preparedness measures have been lacking in the Indian policies.

## COVID-19 and homelessness

WHO suggested a three-pronged strategy to contain the current pandemic, namely social distancing, hand, and respiratory hygiene. These have proved to be effective in community containment in certain worst-affected parts of China (Wilder-Smith & Freedman, 2020). While the implementation of these measures and public compliance to them itself is fraught with challenges, what about the homeless population who have restricted access to fundamental human rights including a roof over their heads and health care? Under homeless conditions where minimum personal and environmental cleansing facilities are unavailable, having them use precautionary sanitary measures is a far-fetched idea. Here are few factors adding to their susceptibility:

**Social isolation and its applicability:** Following the complete lockdown in the country, the ‘stay at home’ directive becomes an oxymoron when it comes to the 1.77 million homeless Indians (Goel & Chowdhary, 2017). While self-isolation and home quarantine are to be maintained to ‘flatten’ the graph before it reaches the next stage, what option do the homeless people have? When a family of more than 10 struggles to find accommodation in a limited area on the streets, it is but fanciful to mention social distancing. There is no doubt that physical proximity spreads contagion. The government has not issued any directive as of now on dealing with this issue. On the other hand, to maintain the sanctity of the lockdown, there have been reports of forced displacement (Lewis et al., 2020).
**Poor living conditions leading to poor immunity:** Homeless individuals are more prone to many factors including malnutrition to perhaps lowered immunity to catch infectious agents including COVID-19 due to overcrowding, lack of public hygiene, inadequate waste disposal, weather extremes, contamination, increased prevalence of infections, and substance abuse with overall poorer quality of physical and mental health.
**Homeless migrant laborers and COVID-19:** In India, the majority of the homeless population are daily wage workers, primarily migrant laborers, or beggars (excluding the street children or those with mental illness). With a lockdown activated for 21 days, most of them have been rendered penniless, with not even the option to go back to their native towns or villages. Thousands of such migrant laborers are stranded on the roadsides throughout India. While a few initiatives are being taken to provide them with food and shelter, it is again implausible to maintain any form of social-isolation or hygiene in such scenarios. Stigma and marginalization have also been rampant, when they have been blamed to be responsible for the spread of infection in various parts. There have been instances of ‘hosing’ of the migrant and homeless laborers in an attempt to ‘disinfect’ them that goes against ethics and human dignity.
**Lack of information and testing**: There is hardly any information on COVID-19 for this homeless who may be illiterate or poorly educated . They are more concerned about dying of hunger before COVID-19 affects them. This is compounded by their lack of testing, leading to under-detection and neglect. The transient nature of homeless population is adds to the chaos. Early case detection and contact tracing, two important strategies in the COVID-19 pandemic are thus ‘myths’ in this population.
**Lack of responsibility:** The homeless have always been the ‘crowd of neglect’, not accounted for either by the varied social classes or the administration. Consequently, the health-policy measures rarely touch this ‘vestigial’ but a substantial section of the population.

## Policy implications: the way forward

Professionals across the country are working at a breakneck pace to win the race against COVID-19. Without undermining the efforts that have been taken by the Governments, medical professionals, and policymakers to prevent the outbreak in a populous country like India, it might be safely said that very little has been done to save the homeless population from the ongoing pandemic crisis. With COVID-19 spreading fast, India, as a nation, needs to act fast with a focus on this section of the population as well. Indeed, while the homeless individuals have been rendered resourceless due to the lockdown, many governmental and nongovernmental initiatives have been undertaken to provide them with shelter and food, but that would not save the spread of the virus. A coordinated effort from all the stakeholders – government, community mental health services – as well as collaboration with organizations working for homeless, is required. While quarantine and social isolation are difficult to maintain among hundreds of thousands of homeless people (at present the number has increased with migrant workers rendered homeless), some critical action steps are outlined below:

**Providing shelter and food:** For these basic living amenities, the government can utilize school and college buildings, community halls, and so on, to arrange the temporary shelters. All the existing night-shelters throughout the country should be made available for 24/7 support. The government has already made resources available to supply food to the homeless. Stakeholders should be vigilant on the proper utilization of the allocated resources. Transient shelters can be modified based on their needs. Similarly they need to get equal access to the quarantine facilities, if need arises.
**Providing essential resources to maintain hygiene:** Even when it is not possible to maintain absolute social distancing, it is necessary to maintain personal hygiene, thus an adequate supply of basic hygiene essentials like soap, masks, and disinfectants must be made available.
**Testing for the virus among the vulnerable:** An increased number of testing within those who are identified as vulnerable is required. Those tested positive then need to be quarantined for the stipulated period and appropriate health care initiated. The need for testing should be repeatedly emphasized upon among their families.
**Accessibility to mental health support:** Of the existing homeless population, 20–25% suffer from some form of mental illness and substance abuse (Gopikumar et al., 2015). Holistic care to manage anxiety and stress related to the pandemic, uncertainty, lack of a job, lockdown must be put in place. Supportive psychological interventions are vital.
**Educating the homeless**: Knowledge, attitude, and practices need to be improved through community programs. Necessary precautionary measures need to be explained in their language and doubts clarified. All the Universal Human Rights and basic self-dignity applies them as well, which need to be ensured by all possible means.

The Indian pandemic act of 1897 has not been updated in keeping with the recent developments of infectious diseases and anyhow the homeless population are not a part of it. As we struggle to prevent community transmission, this crucial but vulnerable population might hold the key to our success. The denial or neglect of the other sections of the society towards them, will not necessarily physically ‘exclude’ them from the mainstream and make them immune to the effects of such a contagious virus. Even one infection in the overcrowded street-sides or slums can rapidly fuel the spread of this outbreak, taking it well beyond control. Policy implications and administrative measures need to ensure that the homeless population is rehabilitated in basic dignity, empowered, and helped against the infection just like others. The spread of a pandemic has bidirectional relationships with the ‘pandemic’ of homelessness as well, which, if neglected, will make all our containment efforts futile, especially in India. Such measures also need to be incorporated in relevant policy-making for dealing with such futuristic biological disasters. COVID-19 gives us just another short-lasting opportunity to understand that in context of public health.
